# Categorisation of Antimicrobial Use in Fijian Livestock Production Systems

**DOI:** 10.3390/antibiotics11030294

**Published:** 2022-02-23

**Authors:** Xavier Khan, Caroline Rymer, Partha Ray, Rosemary Lim

**Affiliations:** 1 Department of Animal Sciences, School of Agriculture, Policy and Development, University of Reading, P.O. Box 237, Reading RG6 6EU, UK; c.rymer@reading.ac.uk (C.R.); patha.ray@tnc.org (P.R.); 2 The Nature Conservancy, 4245 North Fairfax Drive, Suite 100, Arlington, VA 22203, USA; 3 Reading School of Pharmacy, School of Chemistry, Food & Pharmacy, University of Reading, Whiteknights, Reading RG6 6DZ, UK; r.h.m.lim@reading.ac.uk

**Keywords:** antibiotics, anthelmintics, prudent use, imprudent use, livestock production systems, Fiji

## Abstract

Antimicrobial resistance (AMR) is a major global threat to human and animal health. The use of antimicrobials in the livestock sector is considered to contribute to AMR. Therefore, a reduction in and prudent use of antimicrobials in livestock production systems have been advocated. This cross-sectional survey aimed to investigate the extent of imprudent antimicrobial use (AMU) and to determine whether the AMU practice was affected by either the farming system or species of farmed livestock in the largest island (Viti Levu) of Fiji. A total of 276 livestock enterprises were surveyed and antimicrobials were used on 309 occasions over 90 days. Overall, in 298 of 309 (96%) incidents, antimicrobials were used imprudently, comprising antibiotics, 160 of 170 (94%) and anthelmintics, 138 of 139 (99%). Prudent use of antibiotics was associated with commercial farming systems (X^2^ = 13, *p* = 0.001), but no association was observed with anthelmintic use (*p* > 0.05). Imprudent antibiotic use was associated with dairy (OR = 7.6, CI = 1.41, 41.57, *p* = 0.018) followed by layer and beef (*p* > 0.05) compared to broiler enterprises. Imprudent AMU was more common in the backyard and semi-commercial enterprises compared to commercial broiler enterprises. Policies promoting the prudent use of antimicrobials in Fiji should focus on smaller livestock production systems and enterprises.

## 1. Introduction

Antimicrobial resistance (AMR) is a significant global threat to human and animal health [1]. Antimicrobial use (AMU) in the livestock sector has been considered to contribute to the AMR issue [1,2]. Therefore, a reduction and a prudent use of antimicrobials in livestock production systems have been advocated [1,2,3]. Food of animal origin is produced using traditional systems in non-commercial farming settings for food and socio-economic security worldwide [4,5,6]. However, in recent times, increasing demand for foods of animal origin (meat, milk, and eggs) has contributed to the intensification and commercialisation of livestock production systems locally and globally [7,8,9]. Larger flocks/herds of animals are produced in smaller confinements (sheds, cages, and paddocks) and in a shorter duration than traditional, extensive and free-range systems [6,8,10]. Backyard farming systems continue to produce livestock for domestic consumption, while semi-commercial farmers who are market-orientated produce livestock for domestic consumption and sale [11,12].

Higher flock/herd density with compromised farm biosecurity infrastructure results in higher chances of transmission of diseases amongst flocks/herds; therefore, striking a balance between increasing production and managing farm biosecurity risks has been a challenge faced by livestock farmers [13,14]. As part of a farm-level biosecurity risk management strategy, antimicrobials including antibiotics and anthelmintics (as well as other agents such as vaccines, medicated feed, nutraceuticals, and other herbal preparations) have been used to reduce the risk of microbial infection in the agri-food value chain [15,16,17,18,19,20,21,22,23]. Antimicrobials have traditionally been used to treat diseases in livestock; however, antimicrobials have also been used prophylactically in flocks/herds of animals and for growth promotion [16,24,25]. The prophylactic use may be predominant in commercial systems due to higher stock density; however, it is presumed that AMU for growth promotion may be more common in systems that have fewer veterinary interventions [22,26,27]. Unnecessary and imprudent use of antimicrobials in livestock production systems is of grave concern due to the risk of emergence and transmission of antimicrobial resistance (AMR) genes via the agri-food value chains to humans [28,29]. 

The World Organisation for Animal Health (OIE) discourages unnecessary and long-term use of antimicrobials in farmed livestock and advocate the administration of antimicrobials under the supervision of a qualified veterinarian [30,31]. In addition, the use of World Health Organisation (WHO) classified critically important antimicrobials in livestock production systems is prohibited and considered imprudent [32,33]. The imprudent use of antimicrobials has been reported in developing countries, predominantly in the backyard and semi-commercial farming systems, due to lack of access to veterinarians leading to self-prescribing [5,14,16,24]. However, such AMU practices in Fiji are currently unknown. In the United Kingdom (UK), the Veterinary Medicines Directorate (VMD) produced a code of practice on the responsible use of animal medicines in farm animals for livestock keepers, including guidelines for the access, usage and recording of AMU [34]. At a sectoral level, the Responsible Use of Medicines in Agriculture (RUMA) set guidelines for beef, dairy, broiler, layer and other enterprises [35]. Policies on responsible antibiotic use for farm livestock under cascade were also established in the UK, where veterinarians are permitted to prescribe medicines unauthorised for use in livestock [36], but this has not been the case in Fiji. In Fiji, the shortage of veterinary professionals, lack of legislation restricting AMU in livestock and standard therapeutic guidelines have been reported [37]. In addition, Fijian veterinary legislation is outdated since the current one dates to 1956 [38], while the antibiotics and anthelmintics for use in livestock remain unclassified in the current Medicinal Products Act [39]. On the other hand, the existence of a veterinary authority (a standard-setting authority similar to the VMD and BVA) remains unclear, while the legislation targeting antimicrobial residue levels in animal products only outlines standards on milk and milk products, excluding all other animal products [38,39,40]. Moreover, AMR in the Fijian health sector has also been reported [37].

In the UK, the BVA, in collaboration with VMD, set good practice guidelines for the use of veterinary medicines to assist veterinarians, pharmacists and suitability qualified personnel [41,42]. The VMD guidelines also include off label and cascade use of antibiotics which is only permitted for use under the supervision of veterinarians [36,42]. Hence, deviation from the set regulatory framework and classification on prescribing and dispensing of the antimicrobials is considered imprudent in the UK [43,44]. Antimicrobial legal categories and classification in other South Pacific countries such as Australia and New Zealand are similar to the UK [43,44,45]. However, the Fijian jurisdiction does not define such specific legal categories and authorisation of veterinary medicines [39]. Our recent study demonstrated moderately high use of antimicrobials which varied by systems and enterprises; however, the quantity of antimicrobials used does not demonstrate whether the antimicrobials were used prudently [46]; therefore, this study aimed to investigate the extent of imprudent AMU in Fiji, and to determine whether this was affected by either farming system or species of farmed livestock.

## 2. Results

### 2.1. Characteristics of Antimicrobials Used 

The characteristics of AMU in farm enterprises are presented in Table S1. Veterinary antimicrobials were used in 306 of the 309 (99%) incidents, but on three incidents (1%), human antimicrobials were used, which are prohibited for use in food producing livestock. A little over half of the antimicrobials used were antibiotics (*n* = 170, 55%). Most of the antimicrobials were administered as an oral solution (*n* = 227, 73%), while a smaller proportion were administered orally as powders (*n* = 62, 20%). All powdered formulations for oral use were reconstituted and administered in drinking water. Most antimicrobials contained a single active pharmaceutical ingredient (*n* = 251, 81%). The majority of administrations were in flocks of poultry or herds of cattle (*n* = 253, 82%) rather than to an individual bird or cow, and 87% (268 of 309) of all administrations were self-prescribed (prescription and administration of antimicrobials to livestock on the farms) by the farmer or farm manager. Antimicrobials were mainly purchased from agricultural or veterinary clinics (*n* = 263, 85%) operated by livestock officers (presumably with tropical agriculture qualifications), referred to as para-veterinarians in the Fijian context. 

The most commonly used antimicrobial was anthelmintic of the imidazothiazole derivative class (*n* = 72, 31%) followed by the antibiotic β-lactams (*n* = 81, 26%) and tetracyclines (*n* = 58, 19%) while other antibiotic classes were less frequently used (<10%). A larger proportion of antimicrobials were administered to dairy calves (*n* = 82, 27%) followed by lactating cows (*n* = 53, 17%), bull calves (*n* = 42, 14%) laying hens (*n* = 30, 10%) and broiler breeding birds (*n* = 25, 8%).

### 2.2. Imprudent Antimicrobial Use Categorisation

The categorisation of AMU practice in livestock enterprises is presented in Table 1. According to the first criterion in the decision-making tree (step 1), all anthelmintics (100%) and almost all antibiotics (98%) passed the step because veterinary antimicrobials were used in 306 of 309 (99%) occasions. The 3 of 309 (1%) uses were imprudent because they were non-veterinary antibiotics. Veterinary anthelmintics were used on 139 of 306 incidents and antibiotics on 167 of 306 incidents (step 2). However, since antibiotics are classified as POM-V and anthelmintics as POM-VPS, antimicrobials were mostly used in an imprudent way, as assessed in step 3 (288 of 306, or 94% of occasions). On this criterion, antibiotics were not prescribed by an authorised prescriber on 156 of 167 (93%) incidents and anthelmintics on 132 of 139 (95%) incidents. In step 4, 18 of 306 (6%) incidents prescribed by the authorised prescriber in step 3 were administered to the target species specified on the label and as per their market authorisation (step 4).

Based on our evaluation, we found that in 6 of 18 incidents, antimicrobials which we classified as imprudent use were prescribed by an authorised prescriber in step 3 and approved target species in step 4, but we were unaware of the pre-existing condition being treated and therefore classified the use as imprudent in step 5. Since antibiotics should only be used in cascade as per our set framework (Table 5), 11 of 167 (7%) incidents (step 3) antibiotics used were categorised as cascade use in step 6.

On 18 occasions, antimicrobials were administered to authorised target species (step 4); however, only in 5 of 7 incidents were anthelmintics used prudently, and in 7 out of 11 incidents, antibiotics were used prudently. Since antibiotics are prescribed in cascade and all 11 of the 167 incidents were prescribed by the authorised prescriber in step 3, we considered all 11 for cascade use. Maintaining AMU records is essential for antimicrobial stewardship (AMS) programmes. Only 1 out of 5 incidents of anthelmintics were recorded (thus used prudently) whilst 10 out of 11 incidents of antibiotic use were recorded (i.e., prudent) (step 7).

Overall, in 298 of 309 (96%) incidents, antimicrobials were used imprudently. Antibiotics on 160 of 170 (94%) incidents and anthelmintics on 138 of 139 (99%) incidents were used imprudently. The practice of AMU (imprudent, prudent) was associated with the antimicrobial type (anthelmintic, antibiotic), *p* = 0.026, with antibiotic use being marginally more prudent compared to anthelmintics (Table 2).

### 2.3. Description of the Types of Antimicrobials Used

OIE classified veterinary critically important antimicrobials (antibiotics only) were used on 55% of 309 incidents, while WHO categorised high priority critically important antimicrobials (macrolides, beta-lactams and third and fourth generation cephalosporins) were used on 3% of occasions (refer Table S1). Antimicrobial agents used as antiparasitic agents (45%) and antimicrobial agents for systemic use (38%) dominated the groups of antimicrobials used. According to the VMP classification, 57% of antimicrobials used were POM-V, and 42% were POM-VPS. Almost half of the administrations (48%) were classified as therapeutic use, while the remaining half was for prophylactic purposes (37%) and growth promotion (15%). Although the prescriber was one of the main prerequisites for categorisation of imprudent use, overall, in 36 incidents (12%), AMU was off label or antimicrobials were used in unauthorised target species, and there were only three incidents (1%) when prohibited antimicrobials were used (Table 3).

**Figure 1:**
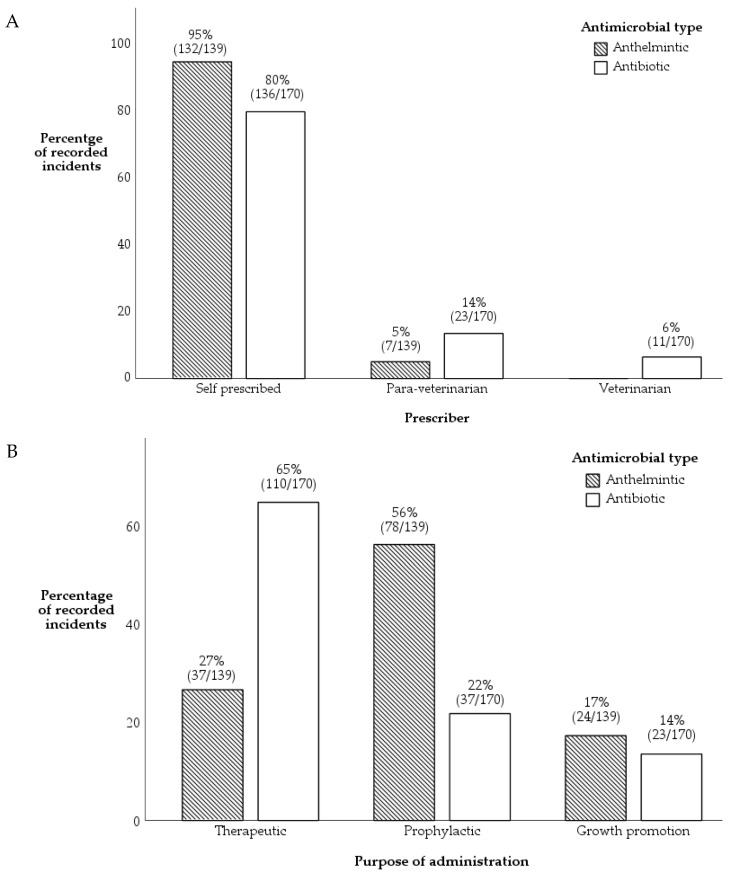
Association of 309 incidents where antimicrobials were used (anthelmintics *n* = 139, antibiotics *n* = 170) with (**A**). prescribing pattern (Fisher’s exact test, *p* < 0.001), and (**B**). purpose of administration (X^2^ = 48, *p* < 0.001) in 276 enterprises located in Central and Western division of Viti Levu, Fiji.

In most incidents, farmers self-prescribed anthelmintics (95%) and antibiotics (80%). Para-veterinarians prescribed antibiotics on only 14% of occasions, and veterinarians on only 6% of occasions. There was an association between the type of antimicrobial used and the prescriber, with veterinarians prescribing marginally more antibiotics than anthelmintics (*p* < 0.001) (Figure 1A). 

Most antibiotics were used therapeutically (65% of occasions), over a fifth (22%) were used prophylactically and 14% as growth promoters. The principal use of anthelmintics was for prophylactic purposes (56%), but on 17% of incidents, anthelmintics were used for growth promotion. There was a significant association between antimicrobial type and purpose of administration type, with greater usage of anthelmintics for prophylaxis (X^2^ = 48, *p* < 0.001, Figure 1B).

**Figure 2:**
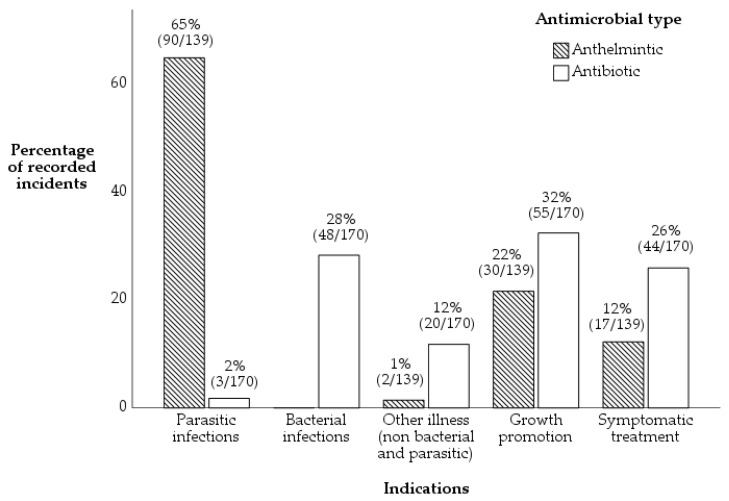
Association between 309 incidents where antimicrobials were used (anthelmintics *n* = 139, antibiotics *n* = 170) and indication of use on 276 enterprises located in the Central and Western division of Viti Levu, Fiji. (Chi-square statistics X^2^ = 162, *p* < 0.001).

Anthelmintics were used in just over 60% of incidents for parasitic infections, while antibiotics were used in a little over 30% of incidents for growth promotion. Anthelmintic and antibiotics were also used for symptomatic treatment (12% and 26%, respectively). The AMU practice was associated with indications of use, with anthelmintics being mainly used for parasitic infections (X^2^ = 162, *p* < 0.001, Figure 2).

### 2.4. Prescription of Antimicrobials in Enterprises and Farming Systems 

**Figure 3:**
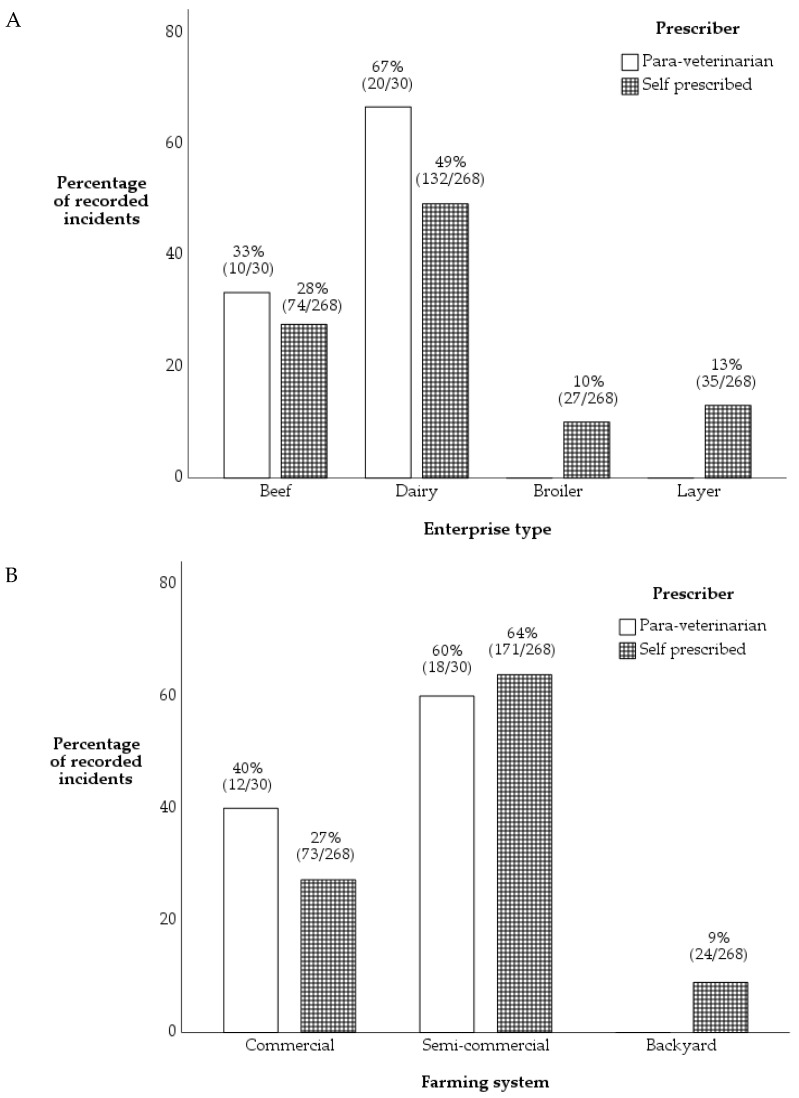
Association between 298 of 309 incidents where antimicrobials were prescribed (para-veterinarians *n* = 30, self-prescribed *n* = 268) and (**A**). different enterprises (Fisherman’s exact test, *p* = 0.017) and (**B**). different farming systems (Fisherman’s exact test, *p* = 0.111) located in the Central and Western divisions of Viti Levu, Fiji. Veterinarians only prescribed in commercial broiler enterprises (*n* = 11 incidents) and were excluded from the analysis.

Antimicrobials were not prescribed by veterinarians (Refer Table S1, 298 of 309 incidents) except in broiler enterprises (100%, 11 of 11 incidents). Para-veterinarians were most likely to be used in dairy enterprises (67%, 20 of 30 incidents) and did not prescribe antimicrobials in poultry enterprises (*p* = 0.017, Figure 3A). Farmers self-prescribed antimicrobials in all enterprises, although this was less common in broiler and layer enterprises (10% and 13%, respectively). Farmers self-prescribed antimicrobials most commonly in dairy enterprises (49%, 132 of 268 incidents). 

Veterinarians only prescribed antimicrobials (refer to Table S1, 298 of 309 incidents) in commercial farming systems (100%, 11 of 11 incidents) while para-veterinarians mostly prescribed in semi-commercial farming systems (60%, 18 of 30 incidents). Self-prescribing was also common in semi-commercial systems (64%) and commercial farming systems (40%). Veterinarians and para-veterinarians did not prescribe any antimicrobials for use in backyard farming systems. However, there was no statistically significant association between prescriber and farming system (*p* = 0.111, Figure 3B).

### 2.5. Associations and Logistic Regression Modelling of Farming System and Enterprise Type with AMU Practice 

Amongst the different farming systems, antibiotics were used imprudently in more incidents in the semi-commercial farming system (98%). The situation was only marginally better in the backyard (92%) and commercial (76%) farming systems. There was an association between farming systems and AMU practice where prudent use mainly was in commercial farming systems (X^2^ = 13, *p* = 0.001). 

The antibiotics were administered imprudently on more incidents in dairy (96%) followed by layer (95%) and beef (94%); however, the situation was better in broiler enterprises (75%). There was an association between AMU practice and enterprise type (X^2^ = 10, *p* = 0.022), where broiler enterprises were more likely to use antimicrobials prudently. 

All anthelmintics administered in the backyard and semi-commercial farming systems were used imprudently. Anthelmintic use was imprudent in beef, dairy and layer enterprises and only slightly better in broiler enterprises. There was no association between anthelmintic use practice and farming systems and enterprise types (*p* > 0.05). 

Imprudent use was also more likely to be practiced in dairy enterprises (OR = 7.6, CI = 1.41, 41.57, *p* = 0.018, Table 4) followed by layer enterprises and beef enterprises compared to broiler enterprises; however, our finding was statistically insignificant for layer and beef enterprises (*p* > 0.05).

## 3. Discussion

The present study, to our knowledge, is the first study categorising AMU in livestock production systems in Fiji. The study revealed that in 96% of incidents (298 of 309), antimicrobials were used imprudently on 276 enterprises. The evaluation revealed that 94% (160 of 170 incidents) of antibiotic (POM-V) use and 99% (138 of 139 incidents) of anthelmintic (POM-VPS) use were categorised as imprudent. Although antimicrobials were purchased from veterinary clinics, presumably operated by para-veterinarians, the prescription and administration of antimicrobials to livestock on the farms were done by the farmers (95% self-prescribed). The results also suggest that most POM-V and POM-VPS were sold to farmers without prescription, concurring with the findings of studies in other developing countries [24,47].

The results also suggest that 12% of antimicrobials were administered to unauthorised target species, thus deviating from the market authorisation and the label. Given that 87% of antimicrobials were used in authorised target species, we presume that these antimicrobials were used correctly; used as per indications given in the market authorisation and the labels of the antimicrobials (see Table 3). The indications for which the antimicrobials were used were at the discretion of the farmers. Nonetheless, the actual administration on the farm depends on the farmers’ decision-making process where the farmers’ intention, attitude and available resources play a fundamental role in deciding the AMU [48], which differs from the UK, where the antibiotics are only prescribed by the veterinarians while anthelmintics by a pharmacist or suitably qualified person or a veterinarian [49].

On the other hand, the unauthorised prescription and administration of antimicrobials are of grave concern. There is a high chance of incorrect dosing, non-compliance to the correct duration of use, and off-label use resulting in imprudent use similarly reported in studies in other countries [47]. Europe and the UK banned the use of antibiotics for growth promotion [50,51]; however, antibiotics continue to be used for growth promotion in the Asian Pacific region [16,52]. The current study’s findings confirmed that antimicrobials were used for growth promotion and were also administered prophylactically in livestock farms in Fiji. We presume there may be chances of justified and right intentions for using the antimicrobials in livestock to mitigate farm biosecurity risks [26]. However, using antimicrobials without consulting veterinary professionals poses a greater chance of antimicrobials being used imprudently [48]. We also believe that some imprudent use may be due to a lack of knowledge and understanding of antimicrobials, as participants reported using anthelmintics and antibiotics to treat similar diseases. In was beyond the scope of this study to explain the motivations behind the AMU practice.

Although antimicrobials were used imprudently in the vast majority of incidents (96%), most of them were used on authorised species (Table 1, Step 4). This finding further infers that although there was easy access to antimicrobials, only 12% of antimicrobials were administered to unauthorised species at the farm level (Table 3). However, we could not elucidate if antimicrobials were under or overdosed; therefore, randomised longitudinal quantitative studies that collect the body weights of animals and the ailments treated at the point of treatment with the antimicrobials would be more appropriate. Subsequently, the individual dose and course doses according to the indication for use could be more accurately estimated so that the under or overuse of antimicrobials in the different enterprises could be evaluated.

In the current study, antimicrobials were usually administered to the flock or herd rather than individual animals, which suggested that antimicrobials might have been used on clinically healthy animals. Prophylactic use of antibiotics in animals has been discouraged by OIE [53], but to maintain biosecurity, prophylactic use may be justifiable for economic and welfare reasons. Nevertheless, the prophylactic use of anthelmintics has been found to be beneficial in reducing the number of macroparasite infections reported in other studies [54]. Some studies have reported that one of the motivations for implementing biosecurity was increasing profit through higher farm production [15,55]. Therefore, we suggest further studies to explore and understand the motivations for implementing biosecurity measures such as prophylactic use of antimicrobials in flocks and herds of cattle and poultry. We further suggest exploring the farm biosecurity risk mitigation strategies employed on farms, reducing the incidence and transmission of diseases, thus reducing the need for antimicrobials. This will enable the development and recommendation of more uniform sectoral and enterprise-level risk management strategies. Exposing clinically healthy herds and flocks of animals to antimicrobials may further contribute to the risk of AMR, as demonstrated in other studies [29]. Therefore, in the Fijian jurisdiction, we suggest that care must be taken when administering antimicrobials at the herd and flock level. In addition, all administrations should be executed in consultation and under the supervision of suitably qualified veterinarians following the guidance of the WHO, OIE and FAO [2,3]. We also found that veterinary critically important antibiotics [33] and antibiotics critically important for human medicine [32], such as tetracyclines and β-lactam penicillin, were commonly used and were similarly reported in other developing countries [22,51,52].

Our results also revealed that antibiotics were used under the cascade, and the record-keeping of AMU was inconsistent. We could not establish a general understanding of the importance of maintaining AMU records amongst the livestock farmers and veterinarians. Evaluating and demonstrating an understanding of the decision-making process for cascade use of antibiotics could not be established; however, the cascade use of antibiotics by farmers, without consultation and supervision of veterinarians, is alarming. Contrarily, in the UK, antibiotics can only be used and prescribed by veterinarians under the cascade, adhering to stringent regulatory requirements [36,49]. Therefore, the development and implementation of regulatory frameworks on the cascade use of antibiotics similar to the UK [41,56] would assist the Fijian livestock production sector. In addition, we were also unable to assess the role of the para-veterinarians in veterinary service delivery and AMU decision-making process; therefore, it is crucial to clearly define the roles of para-veterinarians in the legislative frameworks in Fiji so that all POM-VPS (anthelmintics) are appropriately prescribed by para-veterinarians and other professionals as practiced in the UK and other countries [30,36,41]. Given that we were unable to precisely evaluate the roles of the para-veterinarians and veterinarians at large in veterinary service delivery and livestock production, we suggest further studies exploring and understanding the knowledge, attitude and behaviour of the veterinarian and para-veterinarian towards antimicrobial prescribing, AMU and veterinary practice so that policy recommendations could be made to improve veterinary services and strengthen AMS programmes.

Based on the indications of use, antibiotics were used most frequently for growth promotion followed by treatment for bacterial infections and symptomatic treatment; however, antibiotics being used for the treatment of other illness (non-bacterial and parasitic) is of grave concern since such use is contraindicated in the market authorisation and label of the antibiotics. The use of antibiotics for bacterial infections may be justified due to its compliance to market authorisation and label; however, all other uses (parasitic infections, growth promotion, symptomatic treatment and non-bacterial and parasitic illness) described in Figure 2 are considered as imprudent use and contribute to the growing risks of AMR as reported in another study [29].

Our findings revealed that antibiotics were used therapeutically (65%) and anthelmintics prophylactically (56%) (Figure 1B), but the indications of use (Figure 2) showed that a higher proportion of anthelmintics were used (correctly) for parasitic infections compared to antibiotics which were used for growth promotion. Our study also revealed that antimicrobials were used mainly in the early phase of livestock production (Table S1), probably to prevent animal mortality which is more prevalent when the animal is younger [5,18] and to ensure the sustainability of production, which would serve as an income source to the household [6]. Our results show that the percentage of imprudent AMU was higher in semi-commercial and backyard systems. The high use of antimicrobials may contribute to the development of AMR [29] and higher chances of antimicrobial residues being found in food of animal origins as reported in other studies [25,57], therefore contributing risks to all in the agri-food value chain. Given that most enterprises raised livestock in the backyard and semi-commercial farming systems (Table 4), there is considerable reliance for Fijians on the semi-commercial and backyard farming systems despite the commercial farming system being the primary source of food in the agri-food chain, similarly demonstrated in other studies [6]. Therefore, further studies investigating antimicrobial residue levels in beef, milk, poultry meat and eggs are required, so that antimicrobial residue limits could be established and unnecessary exposure of antimicrobials to Fijians via the agri-food value chain could be minimised. Furthermore, understanding of the drivers for AMU practice in all farming systems over a prolonged duration is required so that necessary policies targeting behavioural interventions could be recommended for incorporation in AMS programmes.

There was a more prudent use of antimicrobials in commercial farming systems, and we presume that the imprudent use of antimicrobials in the backyard and semi-commercial farming system may be due to lack of accessibility to veterinary services, compromised farm biosecurity infrastructure and lack of knowledge and understanding on antimicrobials and AMR, which have also been reported in other studies [8,10,12,14,48]. Our evaluation of prescribing patterns in farming systems and enterprises revealed that veterinarians only prescribed antimicrobials in commercial farming systems and broiler only enterprises. We believe this is due to the easy access of in-house veterinarians in commercial enterprises. In addition, commercial broiler farmers are financially capable of accessing veterinary services considering the financial investments and mitigating financial losses that can result from compromised farm biosecurity [15,58]. Therefore, improving the veterinary services by recruiting more qualified veterinarians and creating training opportunities locally may improve veterinary services to all farmers resulting in POM-V (antibiotics) only being prescribed by veterinarians. This will also enable easy access of veterinarians by semi-commercial and backyard farming systems as well as other specialists and mixed enterprises. We presume improving veterinary services and engaging farmers and veterinarians in AMS programmes may improve and promote prudent use of antimicrobials.

Since the actual disease or ailments for which antimicrobials were administered was unknown, there may be chances of incorrect interpretation and classification of antimicrobials. However, this present study provides the framework for categorising antimicrobials and can be used in developing countries where information on AMU and AMR is scarce. We were unable to execute the logistic modelling for antibiotic use in different farming systems and anthelmintic use in different farming systems and enterprises due to lack of statistical association and unbalanced model due to unequal representation in all categories; therefore, we suggest equal representation to be considered in the inclusion criteria for future studies so that logistic modelling could be executed.

The high prevalence of imprudent use categorisation may also be due to the framework used, which was based on international standards. In the absence of the Fijian national medicine’s directorate and legal classification of veterinary antimicrobials, using the UK, EU and OIE regulatory framework on veterinary antimicrobials use as a reference point was the best available choice. Since the UK and EU’s food production systems and standards are a robust encompassing regulatory framework targeting every stage of the agri-food value chain, these standards enabled us to best evaluate AMU practice. Additionally, it was not possible to compare imprudent AMU practice in Fiji with other countries due to limited information regarding the categorisation of AMU practice. Nevertheless, despite vast differences in livestock production between developed and developing countries, AMR is a global threat to all countries. Therefore, the applicability of the international standards is well justified as it helped us establish the current situation on the AMU practice in Fiji.

## 4. Materials and Methods

### 4.1. Study Design and Data Collection

A cross-sectional survey was conducted between May and August 2019 on 236 livestock farms comprising 276 enterprises in Fiji’s largest island’s Western and Central divisions (Viti Levu). Livestock farmers and managers were recruited using a purposive and snowball sampling method. This study’s design and data collection method was part of the principal survey published earlier [46]. The AMU dataset from the principal survey was used in this present study for the categorisation of AMU practice.

### 4.2. Data Management and Analysis

In the absence of a Fijian classification system for veterinary antimicrobials, a seven-step framework was developed using the VMD, BVA, ESVAC and OIE guidelines [36,41,56,59,60] to categorise the AMU (antibiotics and anthelmintics) into either prudent or imprudent use (Table 5). We used a similar approach used in the human health sector where imprudent use of antibiotics was defined as either using antibiotics without prescription, incomplete course and non-compliance to instructions of use [51].

All antimicrobials were classified according to their legal distribution category and market authorisation before being categorised into prudent and imprudent use. While antibiotics were classified as Prescription Only Medicine–Veterinarian (POM–V), anthelmintics were considered Prescription Only Medicine–Veterinarian, Pharmacist, Suitably Qualified Person (POM–VPS). In the current study, Suitably Qualified Persons were livestock officers (agriculture veterinary clinics staff and field officers and other non-government livestock officers) since they undertake para-veterinarian duties. The titles (livestock officer and para veterinarian) are used interchangeably in Fiji as there is no prescribed definition and competencies outlined in the current legislative framework.

The seven-step framework included classifying veterinary antimicrobial use into 1. Antimicrobial type, 2. Antimicrobial class and legal distribution category of antimicrobial, 3. Prescriber of antimicrobials, 4. Target species (authorised as per label or market authorisation), 5. Purpose of administration (metaphylactic, prophylactic, therapeutic, and growth promotion), 6. Antibiotics used under the cascade and 7. Maintenance of farm AMU records (Table 5). All antimicrobials administered on different incidents were individually evaluated and categorised into prudent/imprudent use. Since only antibiotics can be prescribed in cascade, Step 6 was only applicable to antibiotics [36,49].

All AMU was categorised into prudent and imprudent use based on the intended therapeutic indications (purpose of use) of use reported by the farmer and farm manager (refer to Box 1).

Box 1 **Therapeutic Indication Classification.**

- All antimicrobials used for deworming were classified as used to treat/prevent parasitic infections.
- All antimicrobials used for mastitis and other infections were classified as used to treat/prevent bacterial infections.
- All antimicrobials used for other illnesses were classified as used to treat/prevent non-bacterial and parasite infections.
- All antimicrobials used for increasing outputs were classified as used for growth promotion.
- All antimicrobials used for gastrointestinal (diarrhoea), respiratory (flu), and viral illness were classified as symptomatic treatment.

All (*n* = 309 incidents) intended therapeutic indications of use were individually evaluated and categorised using the framework (Table 5) by the first author, a doctoral candidate and pharmacist with experience in agro security, food security and one health (XK), and verified by co-authors, an animal scientist with a doctoral degree and extensive experience in animal sciences (poultry)(CR), academic veterinarian and animal scientist with a doctoral degree with extensive experience in animal sciences (cattle) (PR) and one female academic pharmacist with a doctoral degree in medicine use and safety and extensive experience in qualitative research (RL).

Descriptive statistics were used to summarise the categorical variables; the pharmaceutical, pharmacological, clinical, legal category, therapeutic indications of use, prescribing pattern, source, and purpose of administration (herd/flock vs individual, prophylactic vs therapeutic, growth promotion). Subsequently, AMU practice (prudent/imprudent use) by the farming system and enterprise types were also summarised. The percentage of imprudent antibiotic and anthelmintic uses per enterprise (antibiotic used, *n* = 111 and anthelmintics used, *n* = 94) was calculated using the equation below.

(1) $$\mathrm{Percentage}\mathrm{of}\mathrm{imprudent}\mathrm{use}=\frac{\mathrm{Number}\mathrm{of}\mathrm{times}\mathrm{AM}\mathrm{used}\mathrm{imprudently}}{\mathrm{Total}\mathrm{number}\mathrm{of}\mathrm{times}\mathrm{AM}\mathrm{used}\mathrm{per}\mathrm{enterprise}}\times 100$$

where AM is antimicrobial, and the number of times is incidents on which antimicrobials were used.

The percentage of imprudent use was binary coded into prudent (0% = prudent, coded 0) and imprudent (>0–100% = imprudent, coded 1) for antibiotics and anthelmintics.

### 4.3. Statistical Analysis

Data were analysed using IBM SPSS Software V27. The Pearson’s chi-square or Fisher’s exact test as appropriate was used to investigate the association between the dependent binary-coded variable (prudent = 0 and imprudent = 1) with the antimicrobial types used (antibiotic, anthelmintic), farming system and enterprise type.

To evaluate prescribing patterns, access and use of antimicrobials, the chi-square test or Fisher’s exact test as appropriate was also used to investigate the association between antimicrobial types (antibiotic and anthelmintic) and the prescriber of antimicrobials, purpose of administration, and the indication of use (parasitic infections, bacterial infections, other illness, growth promotion, symptomatic treatment). Subsequently, the Fisher’s exact test was also used to investigate the association between prescribers of antimicrobials with farming systems and the enterprise type. The veterinarian prescriber category was excluded from the analysis since they only prescribed in commercial broiler enterprises.

The enterprise type (independent variable) was fitted in the binary logistic regression model with the antibiotic use practice (outcome variable). The Hosmer and Lemeshow test was used to evaluate the model fit. From descriptive analysis, the enterprise with the highest percentage of prudent AMU was set as the reference category; thus, broiler enterprises were selected as the reference category in the modelling. The logistic modelling was not executed for antibiotic use practice in different farming systems due to unequal representation of sample, while the anthelmintic use practice modelling was also not performed due to lack of statistical association. For all analyses, *p*< 0.05 was considered statistically significant.

## 5. Conclusions

This present study suggests that anthelmintics and antibiotics were used imprudently in all enterprises. Imprudent AMU was more common in the backyard and semi-commercial enterprises compared to commercial enterprises. Policies promoting the prudent use of antimicrobials in Fiji should focus on smaller livestock production systems and mixed enterprises. Transformation and improvement of policies on AMU, improving veterinary services and regulating the access, prescribing, and dispensation of antimicrobials is warranted to promote prudent use of antimicrobials at the country level. Concurrently, follow-up studies to understand AMU drivers in Fijian food production systems is essential as information obtained will enable the development of targeted behavioural interventions to promote prudent AMU in livestock production systems.

## Figures and Tables

**Table 1:** Categorisation of 309 incidents where antimicrobials were used in livestock enterprises located in Viti Levu, Fiji.

Steps	AMU Practice	Antimicrobial Type					
		Anthelmintic		Antibiotic		Total	
		*n*	(%)	*n*	(%)	*n*	(%)
1 * (antimicrobial type)	Prudent	139	(100)	167	(98)	306	(99)
	Imprudent	0	(0)	3	(2)	3	(1)
	Total	139	(100)	170	(100)	309	(100)
							
3 (prescriber)	Prudent	7	(5)	11	(7)	18	(6)
	Imprudent	132	(95)	156	(93)	288	(94)
	Total	139	(100)	167	(100)	306	(100)
							
4 (target species)	Prudent	7	(100)	11	(100)	18	(100)
	Imprudent	0	(0)	0	(0)	0	(0)
	Total	7	(100)	11	(100)	18	(100)
							
5 (purpose of administration)	Prudent	5	(71)	7	(64)	12	(67)
	Imprudent	2	(29)	4	(36)	6	(33)
	Total	7	(100)	11	(100)	18	(100)
							
6+ (cascade)	Prudent	n/a	n/a	11	(100)	11	(100)
	Imprudent	n/a	n/a	0	(0)	0	(0)
	Total	n/a	n/a	11	(100)	11	(100)
							
7 (AMU records)	Prudent	1	(20)	10	(91)	11	(69)
	Imprudent	4	(80)	1	(9)	5	(31)
	Total	5	(100)	11	(100)	16	(100)
							
Last step **	Prudent	1	(1)	10	(6)	11	(4)
	Imprudent	138 ^a^	(99)	160 ^b^	(94)	298 ^c^	(96)
	Total	139	(100)	170	(100)	309	(100)

Note: - denotes zero *n* (counts) and % (proportion), * denotes steps as per framework (Table 5) where steps 2a and 2b were verification steps, + denotes step 6, which is only applicable to antibiotics, AMU denotes antimicrobial use, ** last step denotes totals of all steps including human antimicrobials used, ^a^ denotes anthelmintics imprudent sum = step 1 + step 3 + step 4+ step 5 + step 7, ^b^ denotes antibiotics imprudent sum = step 1 + step 3 + step 6 + step 7 (steps 4 and 5 are not applicable as antibiotics are prescribed in cascade), ^c^ denotes antimicrobial imprudent total sum = step 1 + step 3 + step 4 + step 5 + step 6 + step 7, less 4 from step 5 (antibiotics used in cascade).

**Table 2:** The antimicrobial use practice of 309 occasions when antimicrobials were used on 276 enterprises located in Central and Western divisions of Viti Levu, Fiji.

	Antimicrobial Use Practice			
Antimicrobial Type	Imprudent		Prudent	
	*n*	% Observed	*n*	% Observed
Anthelmintic	138	99	1	1
Antibiotic	160	94	10	6

*n* denotes frequency, % denotes percentage observed, Fisher’s Exact Test, *p* = 0.026.

**Table 3:** Classification of antimicrobial formulations used in 309 incidents on 276 livestock enterprises located in Viti Levu, Fiji.

Factor	Sub-Categories	*n*	(%)
OIE classification	Veterinary critically important	170	(55)
	Unclassified antimicrobials	139	(45)
WHO classification	Highly important	162	(52)
	Unclassified antimicrobials	139	(45)
	High priority critically important	8	(3)
ATC ESVAC classification	Antiparasitic use	139	(45)
	Systemic use	117	(38)
	Intestinal use	26	(8)
	Intramammary use	24	(8)
	Systemic use (humans)	3	(1)
VMD legal distribution category	POM-V	279	(57)
	POM-VPS	131	(42)
	Human antimicrobial	3	(1)
Purpose of administration	Therapeutic	148	(48)
	Prophylactic	115	(37)
	Growth promotion	46	(15)
	Metaphylactic	-	-
Use on target species *	Authorised	270	(87)
	Unauthorised	36	(12)
	Prohibited ^+^	3	(1)

Note: * denotes classification based on National Office of Animal Health (NOAH), - denotes zero *n* (count) and % (proportion). OIE, World Organization of Animal Health, WHO, World Health Organization, ATC ESVAC, Anatomical therapeutic classification European Surveillance Veterinary Antimicrobial Consumption project, VMD, Veterinary Medicines Directorate, + prohibited use denotes antimicrobials authorised for human use and prohibited for use in livestock raised for food.

**Table 4:** Summary of association and logistic regression modelling of farming systems and enterprise types with antimicrobial use practice on livestock farms located in Central and Western divisions of Viti Levu, Fiji.

Antimicrobial Type ^+^	Factor	Sub-Categories	*n*	(%)	AMU Practice		Chi-Square Tests	Logistic Regression			
					% Imprudent	% Prudent	χ^2^	*p*-Value	*p*-Value	OR	95% CI
Antibiotic	Farming system	Backyard	12	(11)	92	8	13	0.001	-	-	-
		Semi commercial	65	(59)	98	2			-	-	-
		Commercial	34	(31)	76	24			-	-	-
	Enterprise type	Beef	18	(16)	94	6	10	0.022	0.125	5.67	0.62, 52.09
		Dairy	48	(43)	96	4			0.018	7.60	1.41, 41.57
		Broiler	24	(22)	75	25				1	
		Layer	21	(19)	95	5			0.093	6.66	0.73, 60.81
Anthelmintic	Farming system	Backyard	8	(9)	100	0	-	0.248	-	-	-
		Semi commercial	61	(65)	100	0			-	-	-
		Commercial	25	(27)	96	4			-	-	-
	Enterprise type	Beef	33	(35)	100	0	-	0.837	-	-	-
		Dairy	51	(54)	98	2			-	-	-
		Broiler	1	(1)	100	0			-	-	-
		Layer	9	(10)	100	0			-	-	-

Note: reference category is commercial for farming systems, broiler for enterprise type, *n* denotes the frequency, % denotes percentage, AMU, antimicrobial use, OR denotes odds ratio, CI denotes confidence interval. - denotes logistic regression modelling was not executed as there was no association (Fisher’s exact test, *p* > 0.05) in the anthelmintic model and unbalanced antibiotic model for the farming system, + denotes two models (antibiotic, anthelmintic).

**Table 5:** Framework for categorisation of antimicrobial use practice in livestock farms.

Step	Categories	Description	Procedure
1	Antimicrobial type	Verify if veterinary antimicrobial or human antimicrobial was used.	If veterinary antimicrobial was used, proceed to step 2A; if human antimicrobial was used, use was categorised as imprudent.
2	Antimicrobial class	Classify into class: antibiotics or anthelmintics.	Identify the class of the antimicrobial and then proceed to step 2B.
	Legal distribution categories of veterinary antimicrobials	Classify into either: Authorised Veterinary Medicine–General Sales List (AVM–GSL), Non-Food Animal Veterinarian, Pharmacist, a Suitably qualified person (NFA-VPS), Prescription Only Medicine–Veterinarian, Pharmacist, Suitably Qualified Person (POM–VPS), Prescription Only Medicine–Veterinarian (POM–V).	Identify and classify the veterinary antimicrobial if antibiotics were used and then proceed to step 3. (NOTE: all antibiotics used orally, parenterally and in-feed were classified as POM-V, anthelmintics as POM-VPS, and suitably qualified person (SQP) was a person trained and registered to sell veterinary medicine from agriculture store)
3	Prescriber	Verify the prescriber; POM–V can only be prescribed by a Veterinarian, POM–VPS (Veterinarian, Pharmacist, Suitably qualified person), NFA–VPS (Veterinarian, Pharmacist, Suitably qualified person), AVM–GSL (General, Self-prescribed, Other farmers).	If prescribed by the authorised prescriber, then proceed to step 4; if not, the use was categorised as imprudent. (NOTE: for steps 4 to 7, if prescribed not in accordance to step 3, then the use was categorised as imprudent at all steps)
4	Target species	Verify the species administered with approved target species according to market authorisation (MA) and label (authorised, unauthorised).	If deviated from the MA, label and prescribed by the veterinarian, or prescribed as per the MA, label and by the authorised prescriber, then proceed to step 5; if not, the use was categorised as imprudent.
5	Purpose of administration	Verify the purpose and establish the administration type: Therapeutic, Prophylactic, Metaphylactic, Growth promotion.	If prescribed for growth promotion, then the use was categorised as imprudent. If deviated from the MA, label and prescribed by the veterinarian, or prescribed as per the MA, label and by the authorised prescriber, then proceed to step 6; if not, the use was categorised as imprudent.
6	Cascade use	Verify the use of veterinary antimicrobial and prescriber.	If deviated from the MA, label and prescribed by the veterinarian in steps 4 and 5, then the use was categorised as cascade and then proceed to step 7; if not, the use was categorised as imprudent.
7	Farm AMU records	Verify if records were maintained.	If used under the cascade and maintained the antimicrobial use records, then the use was categorised as prudent; if not, the use was categorised as imprudent.

## Data Availability

The data presented in this study are available on request from the corresponding author.

## Supplementary Materials

The following supporting information can be downloaded at: https://www.mdpi.com/article/10.3390/antibiotics11030294/s1, Table S1: Characteristics of 309 antimicrobials used in 236 livestock farms comprising of 276, enterprises located in Viti Levu, Fiji.

## References

[1] WHO Antimicrobial Resistance. http://www.who.int/en/news-room/fact-sheets/detail/antimicrobial-resistance.

[2] OIE Antimicrobial Resistance (AMR). http://www.oie.int/en/for-the-media/amr/.

[3] WHO, FAO, OIE Taking a Multisectoral, One Health Approach: A Tripartite Guide to Addressing Zoonotic Diseases in Countries. http://www.fao.org/ag/againfo/resources/en/publications/TZG/TZG.htm.

[4] Davidova S., Agunos A., Fredriksson L., Bailey A. (2009). Subsistence and semi-subsistence farming in selected EU new member states. Agric. Econ..

[5] Sultana R., Nahar N., Rimi N.A., Azad S., Islam M.S., Gurley E.S., Luby S.P. (2012). Backyard poultry raising in Bangladesh: A valued resource for the villagers and a setting for zoonotic transmission of avian influenza. A qualitative study. Rural. Remote Health.

[6] Rapsomanikis G. The Economic Lives of Smallholder Farmers. http://www.fao.org/3/i5251e/i5251e.pdf.

[7] Cao Y., Li D. (2013). Impact of increased demand for animal protein products in Asian countries: Implications on global food security. Anim. Front..

[8] Henriksen J., Rota A. (2013). Commercialization of livestock production; towards a profitable and market-oriented smallholder livestock production system. Livest. Res. Rural. Dev..

[9] Henchion M., Hayes M., Mullen A., Fenelon M., Tiwari B. (2017). Future Protein Supply and Demand: Strategies and Factors Influencing a Sustainable Equilibrium. Foods.

[10] Malusi N., Falowo A.B., Idamokoro E.M. (2021). Herd dynamics, production and marketing constraints in the commercialization of cattle across Nguni Cattle Project beneficiaries in Eastern Cape, South Africa. Pastoralism.

[11] Kahan D. Market Oriented Farming. www.fao.org/3/a-i3227e.pdf.

[12] Mariyono J. (2019). Stepping up from subsistence to commercial intensive farming to enhance welfare of farmer households in Indonesia. Asia Pac. Policy Stud..

[13] Keutchatang F.D.P.T., Ntsama I.S.B., Nama G.M., Kansci G. (2021). Biosecurity Practices and Characteristics of Poultry Farms in Three Regions of Cameroon. J. World’s Poult. Res..

[14] Moya S., Chan K.W., Hinchliffe S., Buller H., Espluga J., Benavides B., Diéguez F.J., Yus E., Ciaravino G., Casal J. (2021). Influence on the implementation of biosecurity measures in dairy cattle farms: Communication between veterinarians and dairy farmers. Prev. Vet. Med..

[15] Adeyonu A.G., Otunaiya A.O., Oyawoye E.O., Okeniyi F.A. (2021). Risk perceptions and risk management strategies among poultry farmers in south-west Nigeria. Cogent Soc. Sci..

[16] Hosain M.Z., Kabir S.M.L., Kamal M.M. (2021). Antimicrobial uses for livestock production in developing countries. Vet. World.

[17] Kruse A.B., Nielsen L.R., Alban L. (2018). Herd typologies based on multivariate analysis of biosecurity, productivity, antimicrobial and vaccine use data from Danish sow herds. Prev. Vet. Med..

[18] Hoelzer K., Bielke L., Blake D.P., Cox E., Cutting S.M., Devriendt B., Erlacher-Vindel E., Goossens E., Karaca K., Lemiere S. (2018). Vaccines as alternatives to antibiotics for food producing animals. Part 1: Challenges and needs. Vet. Res..

[19] Zhanteng S., Hongting Z., Zhiming X., Decheng S. (2021). Residue accumulation, distribution, and withdrawal period of sulfamethazine and N-acetylsulfamethazine in poultry waste from broilers. Chemosphere.

[20] Tzamaloukas O., Neofytou M.C., Simitzis P.E. (2021). Application of Olive By-Products in Livestock with Emphasis on Small Ruminants: Implications on Rumen Function, Growth Performance, Milk and Meat Quality. Animals.

[21] Abebe B.A., Chane Teferi S. (2021). Ethnobotanical Study of Medicinal Plants Used to Treat Human and Livestock Ailments in Hulet Eju Enese Woreda, East Gojjam Zone of Amhara Region, Ethiopia. Evid. Based Complementary Altern. Med..

[22] Van Cuong N., Nhung N.T., Nghia N.H., Mai Hoa N.T., Trung N.V., Thwaites G., Carrique-Mas J. (2016). Antimicrobial Consumption in Medicated Feeds in Vietnamese Pig and Poultry Production. Ecohealth.

[23] Edwards L.E., Hemsworth P.H. (2021). The impact of management, husbandry and stockperson decisions on the welfare of laying hens in Australia. Anim. Prod. Sci..

[24] Ayukekbong J.A., Ntemgwa M., Atabe A.N. (2017). The threat of antimicrobial resistance in developing countries: Causes and control strategies. Antimicrob. Resist. Infect. Control..

[25] Bamidele Falowo A., Festus Akimoladun O. (2019). Veterinary Drug Residues in Meat and Meat Products: Occurrence, Detection and Implications. Vet. Med. Pharm..

[26] Raasch S., Postma M., Dewulf J., Stärk K.D.C., grosse Beilage E.J.P.H.M. (2018). Association between antimicrobial usage, biosecurity measures as well as farm performance in German farrow-to-finish farms. Porc. Health Manag..

[27] Murphy C.P., Carson C., Smith B.A., Chapman B., Marrotte J., McCann M., Primeau C., Sharma P., Parmley E.J. (2018). Factors potentially linked with the occurrence of antimicrobial resistance in selected bacteria from cattle, chickens and pigs: A scoping review of publications for use in modelling of antimicrobial resistance (IAM.AMR Project). Zoonoses Public Health.

[28] Rega M., Carmosino I., Bonilauri P., Frascolla V., Vismarra A., Bacci C. (2021). Prevalence of ESβL, AmpC and Colistin-Resistant E. coli in Meat: A Comparison between Pork and Wild Boar. Microorganisms.

[29] Ström G., Boqvist S., Albihn A., Fernström L.L., Andersson Djurfeldt A., Sokerya S., Sothyra T., Magnusson U. (2018). Antimicrobials in small-scale urban pig farming in a lower middle-income country—Arbitrary use and high resistance levels. Antimicrob. Resist. Infect. Control..

[30] EU Guidelines for the Prudent Use of Antimicrobials in Veterinary Medicine. https://ec.europa.eu/health/sites/health/files/antimicrobial_resistance/docs/2015_prudent_use_guidelines_en.pdf.

[31] EMA (2019). Categorisation of Antibiotics in the European Union. Categorisation of Antibiotics Used in Animals Promotes Responsible Use to Protect Public and Animal Health.

[32] WHO (2019). Critically Important Antimicrobials for Human Medicine—6th Revision.

[33] OIE (2007). OIE List of Antimicrobials of Veterinary Importance.

[34] VMD (2014). Code of Practice on the Responsible Use of Animal Medicines on the Farm.

[35] RUMA Measuring Antibiotic Use—Dairy, Beef, Poultry. https://www.ruma.org.uk/measuring-antibiotic-use/.

[36] VMD (2015). The Cascade: Prescribing Unauthorised Medicines.

[37] Loftus M., Stewardson A.J., Naidu R., Coghlan B., Jenney A., Kepas J., Lavu E., Munamua A., Peel T., Sahai V. (2020). Antimicrobial resistance in the Pacific Island countries and territories. BMJ Glob. Health.

[38] Goverment of the Republic Fiji (2012). Veterinary Surgeons Act 1956.

[39] Goverment of the Republic Fiji (2011). Medicinal Products Act 2011.

[40] Goverment of the Republic Fiji (2019). Food Safety Reulations 2009.

[41] BVA (2007). Good Practice Guide on Veterinary Medicines.

[42] BVA (2019). Responsible Use of Antimicrobials in Veterinary Practice: The 7 Point Plan.

[43] Bennani H., Cornelsen L., Stärk K.D.C., Häsler B. (2021). Characterisation and mapping of the surveillance system for antimicrobial resistance and antimicrobial use in the United Kingdom. Vet. Rec..

[44] VMD (2020). Product Information Database.

[45] APVMA Australian Pesticides and Veterinary Medicines Authority. https://apvma.gov.au/.

[46] Khan X., Rymer C., Ray P., Lim R. (2021). Quantification of antimicrobial use in Fijian livestock farms. One Health.

[47] Phares C.A., Danquah A., Atiah K., Agyei F.K., Michael O.-T. (2020). Antibiotics utilization and farmers’ knowledge of its effects on soil ecosystem in the coastal drylands of Ghana. PLoS ONE.

[48] Sadiq M.B., Syed-Hussain S.S., Ramanoon S.Z., Saharee A.A., Ahmad N.I., Mohd Zin N., Khalid S.F., Naseeha D.S., Syahirah A.A., Mansor R. (2018). Knowledge, attitude and perception regarding antimicrobial resistance and usage among ruminant farmers in Selangor, Malaysia. Prev. Vet. Med..

[49] VMD (2014). Responsible Antibiotic Use under the Cascade.

[50] Casewell M., Friis C., Marco E., McMullin P., Phillips I. (2003). The European ban on growth-promoting antibiotics and emerging consequences for human and animal health. J. Antimicrob. Chemoth..

[51] Afari-Asiedu S., Oppong F.B., Tostmann A., Ali Abdulai M., Boamah-Kaali E., Gyaase S., Agyei O., Kinsman J., Hulscher M., Wertheim H.F.L. (2020). Determinants of Inappropriate Antibiotics Use in Rural Central Ghana Using a Mixed Methods Approach. Front. Public Health.

[52] Ting S., Pereira A., Alves A.d.J., Fernandes S., Soares C.d.C., Soares F.J., Henrique O.d.C., Davis S., Yan J., Francis J.R. (2021). Antimicrobial Use in Animals in Timor-Leste Based on Veterinary Antimicrobial Imports between 2016 and 2019. Antibiotics.

[53] OIE (2017). Terrestrial Animal Health Code Chapter 6.10.

[54] Roesel K., Dohoo I., Baumann M., Dione M., Grace D., Clausen P.-H. (2017). Prevalence and risk factors for gastrointestinal parasites in small-scale pig enterprises in Central and Eastern Uganda. Parasitol. Res..

[55] Laanen M., Maes D., Hendriksen C., Gelaude P., De Vliegher S., Rosseel Y., Dewulf J. (2014). Pig, cattle and poultry farmers with a known interest in research have comparable perspectives on disease prevention and on-farm biosecurity. Prev. Vet. Med..

[56] EMA Guidance on Collection and Provision of National Data on Antimicrobial Use by Animal Species/Categories 2018. https://www.ema.europa.eu/en/documents/scientific-guideline/guidance-collection-provision-national-data-antimicrobial-use-animal-species/categories_en.pdf.

[57] Adesiyun A., Offiah N., Lashley V., Seepersadsingh N., Rodrigo S., Georges K. (2005). Prevalence of antimicrobial residues in table eggs in Trinidad. J. Food Prot..

[58] Caudell M.A., Dorado-Garcia A., Eckford S., Creese C., Byarugaba D.K., Afakye K., Chansa-Kabali T., Fasina F.O., Kabali E., Kiambi S. (2020). Towards a bottom-up understanding of antimicrobial use and resistance on the farm: A knowledge, attitudes, and practices survey across livestock systems in five African countries. PLoS ONE.

[59] Teale C., Moulin G. (2012). Prudent use guidelines: A review of existing veterinary guidelines. Rev. Sci. Tech. OIE.

[60] OIE OIE Standards, Guidelines and Resolution on Antimicrobial Resistance and the Use of Antimicrobial Agents. https://web.oie.int/delegateweb/eng/ebook/AF-book-AMR-ANG_FULL.pdf?WAHISPHPSESSID=03152ead00d06990fa9066b7b71fcabc.

