# Supplementary material for: Categorisation of Antimicrobial Use in Fijian Livestock Production Systems
Source: Antibiotics (Basel). 2022 Feb 23;11(3):294. doi: 10.3390/antibiotics11030294 (PMC8944440; doi:10.3390/antibiotics11030294)
Supplement: Supplementary file 1 [file antibiotics-11-00294-s001.zip › antibiotics-1564815-supplementary.pdf]

antibiotics

MDPI

Article

Categorisation of Antimicrobial Use in Fijian Livestock Production Systems

Xavier Khan [1,*], Caroline Rymer [1], Partha Ray [1,2] and Rosemary Lim [3]

[1] Department of Animal Sciences, School of Agriculture, Policy and Development, University of Reading, P O Box 237, Reading RG6 6EU, UK; c.rymer@reading.ac.uk (C.R.); patha.ray@TNC.ORG (P.R.)
[2] The Nature Conservancy, 4245 North Fairfax Drive, Suite 100 Arlington, VA 22203, USA
[3] Reading School of Pharmacy, School of Chemistry, Food & Pharmacy, University of Reading, Whiteknights, Reading RG6 6DZ, UK; r.h.m.lim@reading.ac.uk
* Correspondence: x.r.s.khan@pgr.reading.ac.uk

Supplementary Materials

Table S1. Characteristics of 309 antimicrobials used in 236 livestock farms comprising of 276. enterprises located in Viti Levu, Fiji

Factor	Sub-Categories	n (%)
Antimicrobial type	Veterinary antimicrobial	306 (99)
	Human antimicrobial	3 (1)
Veterinary antimicrobial class	Antibiotic	170 (55)
	Anthelmintic	139 (45)
Pharmaceutical form	Oral solutions	227 (73)
	Injections	40 (13)
	Long-acting injections	18 (6)
	Intramammary lactating cow	17 (6)
	Intramammary dry cow	7 (2)
Number of API	One	251 (81)
	Two	32 (10)
	Three or more	26 (9)
Type of formulation	Liquid	247 (80)
	Oral powder	62 (20)
Administration type	Group (flock/herd)	253 (82)
	Single animal	56 (18)
Prescriber	Self-prescribed	268 (87)
	Para-veterinarian	30 (10)
	Veterinarian	11 (4)
Source	Para-veterinarians (vet clinics)	263 (85)
	Veterinarian	7 (2)
	Over the Counter	10 (3)
	Veterinary Retail company	2 (1)
	Other farmers	1 (<1)
	Contractor	7 (2)
	Dairy Cooperative	5 (2)
	Farmers farm cabinet	2 (1)
	Feed supplier	9 (3)
	Other sources+	3 (1)
Antimicrobial class	*Anthelmintics*	
	Benzimidazole derivatives	43 (14)

	Imidazothiazole derivatives	96	(31)
	Antibiotics		
	Aminoglycosides*	26	(8)
	β-Lactams	81	(26)
	Tetracyclines	58	(19)
	Sulphonamides and Trimethoprim*	1	(<1)
	Macrolides	4	(1)
	Enterprise and production stage		
Livestock type	Beef- fattening cattle	4	(1)
	Breeding cattle	37	(12)
	Calves	42	(14)
	Dairy- lactating cow	53	(17)
	Dry cow	14	(5)
	Heifers	3	(1)
	Calves	82	(27)
	Layer-breeding birds	5	(2)
	Layer hens	30	(10)
	Broiler-breeding birds	25	(8)
	Meat birds	13	(4)

Note * denotes formulation with a combination of different classes of antimicrobials, + denotes sourced online, - denotes no scores, n denotes frequency, % represents percentage, API means active pharmaceutical ingredient, all percentages were rounded off to the nearest decimal point, contractor – commercial poultry processor.
